# Model refinement through high-performance computing: an agent-based HIV example

**DOI:** 10.1186/1745-7580-6-S1-S3

**Published:** 2010-09-27

**Authors:** Dimitri Perrin, Heather J Ruskin, Martin Crane

**Affiliations:** 1 Centre for Scientific Computing & Complex Systems Modelling, Dublin City University, Glasnevin, Dublin 9, Ireland; 2 School of Computing, Dublin City University, Glasnevin, Dublin 9, Ireland; 3 Information Sharing Platform Laboratory, Department of Information Networking, Graduate School of Information Science and Technology Osaka University, 1-5 Yamadaoka, Suita, Osaka 565-0871, Japan

## Abstract

**Background:**

Recent advances in Immunology highlighted the importance of local properties on the overall progression of HIV infection. In particular, the gastrointestinal tract is seen as a key area during early infection, and the massive cell depletion associated with it may influence subsequent disease progression. This motivated the development of a large-scale agent-based model.

**Results:**

Lymph nodes are explicitly implemented, and considerations on parallel computing permit large simulations and the inclusion of local features. The results obtained show that GI tract inclusion in the model leads to an accelerated disease progression, during both the early stages and the long-term evolution, compared to a theoretical, uniform model.

**Conclusions:**

These results confirm the potential of treatment policies currently under investigation, which focus on this region. They also highlight the potential of this modelling framework, incorporating both agent-based and network-based components, in the context of complex systems where scaling-up alone does not result in models providing additional insights.

## Background

There is an increasing trend to investigate biomedical systems through the prism of Complexity Science, (as highlighted, for instance, by recent funding calls). This is, in part, motivated by the significant translational potential of models developed in this context.

When considering the immune response to HIV infection, complexity is a direct consequence of the diversity and multitude of cells involved, their mobility patterns, and the various interactions between these cells and the virus. Recent studies have, however, highlighted another layer of complexity, in the sense that local properties of the immune system influence the overall disease progression. Early infection in the gastrointestinal tract, in particular, is crucial [1].

In this paper, we report on a large-scale agent-based model, with explicit implementation of lymph nodes. This structure is tested on a uniform network of nodes, as well as on a network including local properties, to investigate changes in the dynamics of overall disease progression. This model also serves as a basis for discussion on the potential of similar approaches for other biomedical systems.

Given that the immune system is characterised by emergent properties, it is particularly suited to an agent-based approach, with agents uniquely identified with cells. Several such models have been developed to address questions of interest, (see e.g. [2-4]). In their current form, however, these do not easily permit investigation of the impact of local properties on behaviour of the system as a whole.

These properties may lead given areas to exhibit very distinct patterns: this includes the gastrointestinal tract. Of major importance is the role of this tract in terms of overall immune population: it harbours the majority of the body's lymphocytes, where for instance blood only accounts for a few percent of these [5]. Even more importantly, these cells are in close proximity to the external environment and are, therefore, constantly exposed to countless antigens. This results in two crucial properties: more than 90% of these lymphocytes have a memory phenotype, and the proportion of activated cells is significantly higher [6,7]. With HIV primarily targeting CD4^+^ cells, (activated lymphocytes), these factors mean that there is, typically, a large population of targets, (i.e. potential host cells), for the virus to infect and, therefore, a massive infection in the tract even in the early stages of the infection. The immune response in this specific area is, therefore, also very active. Recently published experimental results show:

• A very rapid and very significant decline in CD4^+^ counts, exceeding 25% after four weeks of infection [1].

• Significant levels of infection and destruction observed even within days of infection for memory CD4^+^ cells [8].

• An increased cell proliferation in response to infection. The cell proliferation marker was found on 80% of intestinal CD4^+^ cells four weeks after infection, as opposed to on less than 10% in healthy patients [1].

Due to this local but substantial depletion of immune cells, the overall cell population is also severely reduced, and this imposes significant pressure on the immune system in terms of memory pool maintenance [9]. It also damages lymphoid tissue architecture, and this hinders the ability to support normal lymphocyte homeostasis and antigen presentation.

Early infection in the gastrointestinal tract has, therefore, become an essential of research against HIV. Exact implications of GI tract infection remain largely unclear, but some interesting progress is being made. At the molecular level, it has been shown that preferential targeting of gut-associated CD4^+^ cells may be due to interactions between viral glycoprotein gp120 and integrin α_4_*β*_7_, which is specific to these cells [10].

At a higher level, restoration of cell populations after the acute phase is also under investigation. Observations highlight a delayed and incomplete restoration of cell populations in chronically infected patients, even for those receiving highly active antiretroviral therapy, (HAART), for more than five years [1]. This standard therapy leads to restoration of cell levels in peripheral blood, but not in the tissue considered. A similar therapy, however, if initiated during primary infection, is effective in restoring cell populations. In this case, restoration is a consequence of cell recirculation and increased *homing* from the periphery of the tract, rather than of local cell proliferation.

To evaluate the potential for such treatment policies, it is crucial to understand, in a first instance, how these locally-altered system properties influence the overall infection progression, a crucial objective of the model presented here. Given the importance of the GI tract in the overall progression and its response to the infection itself and to proposed therapies, it is essential to consider the tract within the whole system, rather than as an independent subject. Using a single-node environment would permit the investigation of some aspects of GI tract dynamics, but would not provide any means to study their impact on the overall immune system. A network representation of the lymph network is, therefore, proposed. Tests are performed on large clusters, suitable for massively multi-agent simulations. Here, we reach an agent count in excess of one billion.

## Results and discussion

### Early stages of infection

To estimate the influence of gastrointestinal tract infection in the early stages of disease progression, two sets of simulations are performed, with and without local specification for nodes associated with the GI tract. Significant differences appear, due to GI tract inclusion. In the latter configuration, as soon as the GI tract is infected, the virus finds large amounts of potential targets, and viral spread is enhanced. After two weeks, most of the network is infected. On the other hand, if the GI tract is not represented, the virus still spreads rapidly through the lymph chain where the infection was initiated, but mostly remains restricted to these areas, and two weeks are not enough to obtain complete infection of the overall system. Another difference is in the proportion of infected cells, once the tract is included: ten days after local infection, twice as many cells are infected in several nodes of the GI tract, amounting to 70% of overall node cell count. This value is in agreement with biological studies, which found peak infection occurred at day 10-11, with a subsequent loss of 60 to 80% of memory cells [8].

GI tract inclusion clearly affects the overall viral spread, but does not alter the variability of this spread. Our hypothesis is that this variability is primarily a consequence of inter-node cell mobility, which is not altered by including local properties in the GI tract.

Similar patterns are observed for simulations on various sizes of networks. Thus the proposed model successfully implements gastrointestinal tract properties. Since such an attempt has not been previously reported, and given the importance of the tract in early disease progression, this is a useful complement to existing models.

### Long-term progression

It is also important to consider the effects of this new model feature on long-term progression results, as reported in Table 1. Including the GI tract reduces the length of the acute phase, which is expected from the results on short-term progression. The variability of this length is not significantly altered, and the small difference seems consistent with the current knowledge on the influence of the GI tract: because of the reaction of the tract to the infection, the overall peak is in part determined by the time the infection reaches the tract, (with a local peak ten days after local infection, as detailed above), which increases variability compared to a “uniform” network. Conversely, since cell depletion is increased in the GI tract, the acute phase is locally shorter, and often ends before the overall acute phase, therefore slightly limiting the overall variability of the latter.

**Table 1:** Long-term disease progression

	Standard network	Including GI tract
Peak in acute phase	6.7 weeks [1.2]	6.1 weeks [1.4]
End of acute phase	9.4 weeks [1.6]	8.9 weeks [1.4]
End of latency period	8.0 years [3.7]	7.8 years [3.7]

Average time to reach specific disease progression milestones, for two model configurations. Standard deviations are indicated in brackets.

With respect to the length of the latency period, values obtained from the simulations and shown in Table 1 are slightly lower than clinical results, but the difference is not significant. Overall, GI tract inclusion reduces the latency period. It is also interesting to note that inclusion also improves the model behaviour, which tends to slightly over-estimate the latency period when scaled-up without GI tract inclusion. This is confirmed by looking at ratio between the peak of acute phase, (*t*), and the end of the latency period, (*T*). The ratio between these two time points, (which are the easiest to observe and quantify in both the simulations and the real system), is just under 70,(~ 69.6). In simulations without GI tract, we obtained *T / t* = 62.3 while, with the tract included, *T / t* = 66.7, which is a significant improvement. Future efforts on refining this GI tract implementation will probably further improve model realism, (and, therefore, this ratio). Our understanding of the model is also that the function used to simulate the input of new cells may now be the limiting factor, and that explicit thymus implementation may significantly enhance the model realism.

### Translational potential

There are a number of examples where, using high-end computing, it is possible to scale up well-known approaches and, as a result, gain new insights into the complex systems under investigation. There are, however, cases where scaling up alone does not provide any answer. Thus, complementary solutions, such as model hybridisation, must be sought.

The model presented here is a typical example, where refinement to the agent-based model derives from the inclusion of network-based elements, (and where scaling-up alone was shown to over-estimate the latency period). Hybridisation has been used in other immune models, (see e.g. shape-space models [11]), but this particular hybrid framework may also be applied to other biomedical systems, such as epidemics. Dynamics of disease spread within a population are of crucial importance in terms of public health, (e.g. monitoring of existing outbreaks, preemptive evaluation of intervention policies). Here, a model would have to deal with millions of people living in modern urban environments, each with a refined social behaviour. To date, most approaches have focused on addressing one aspect only.

Network-based models have been used to investigate social structures and their impact on disease spread, (see e.g. [12]). This relies on the fact that, for most infectious diseases, contact is required for a new infection to occur, (sexually-transmitted infections being obvious examples). Social structures are represented by a network where nodes correspond to individuals (or groups), and edges correspond to social links between these. Infection spread is then implemented as a stochastic propagation over the network [13]. This approach, however, is limited by the size of the networks it requires: these are difficult to obtain from real data, or to generate *ab initio*. More crucially, because they are based on social structures, these models can not account for casual contacts between strangers, (e.g. in crowded areas and public transports), which are crucial in common infectious diseases such as influenza.

Conversely, agent-based approaches are suited to model such infections between strangers, which *emerge* from individual behaviour: an infection between travellers on a bus occurs due to individual choices from each, which lead to them boarding, and not because of any link between them, (as opposed to colleagues who *have* to be in the same office, because of this work relationship). Such a system can be efficiently parallelised, as described above for the immune model. The main limitation, however, is the lack of formal framework to include social complex structures.

Given the limitations of both paradigms, it becomes apparent that the hybrid framework presented here for immune modelling is the most efficient solution, combining elements of both approaches. In particular, network-based concepts can be used to generate socially-realistic populations, while the agent basis can simulate an epidemic outbreak within these. This hybrid framework was successfully translated into the epidemic context, and optimally parallelised, providing a realistic framework on which to investigate disease outbreaks and related policies [14].

Although a social basis for agent-based epidemics has been considered by a number of authors, (see e.g. [15]), the focus has been on using activity generators: the social network is then an emergent property of this generator, rather than an explicit model component that can be easily customised by the user. Due to this, handling several types of links, as needed in our model, had not previously been considered. Moving forward, advances in both hardware and computational techniques permit the investigation of larger, and increasingly complex, systems. A socio-medical was described as an example here, but similar frameworks can be applied to microscopic systems such as Epigenetics, where multiple phenomena interact over multiple scales, at the genetic level [16]. Model hybridisation offers a powerful method to account for all aspects of these systems.

## Conclusions

Recent clinical studies highlighted the specific progression patterns of HIV infection within the gastrointestinal tract. Targeting this tract has been suggested as a promising treatment policy. Several computational immune response models have been developed over the years to gain insight into the dynamics of infection progression and the related immune response. Some models proved very successful, and later also served to evaluate treatment policies such as HAART. However, these models are not designed to account for local properties such as those characteristic of the gastrointestinal tract. Biomedical studies, involving direct tracking of HIV viral genotypes in local microenvironments, also revealed that infected cells releasing virions will only induce infection of local targets [17]. This provides further motivation for a modelling approach accounting for local features. It seems evident that local interactions and cell densities are more important to disease progression and experience than overall cell counts in the body [18]. As a bottom-up approach, the agent-based paradigm offers the best prospects for both detailed local solutions and unique identification of cells.

To investigate these, we developed a large-scale agent-based model, which explicitly implements the lymph nodes and the associated network. This permits a detailed implementation of cell mobility, and the inclusion of local properties for a subgroup of nodes, thus reproducing the GI tract.

For an ideal model, an exhaustive list of cell types, together with large populations of each, is desirable, but this is not possible in a first instance. A choice is required between inclusion of “many cells” or “many cell types”. By focusing on the cell-mediated response and using an optimised parallel implementation, we were able to run simulations involving several hundred lymph nodes and more than one billion agents. This permitted the investigation of the GI tract influence on the overall system, which would not be accessible through a model with more cell types but fewer agents for each population, (as outcomes would be restricted to “local” in this case). Future work includes the implementation of other cell types, such as dendritic cells.

The obtained results confirm the importance of the GI tract, in terms of early infection progression but also in terms of long-term evolution, and provides a basis on which to develop the evaluation of possible treatment policies.

The framework we used, in the biological context, viz. a parallel large-scale agent-based model with local features, could also be adapted to the study of other complex systems, such as human interactions in socioeconomic systems.

## Methods

An agent-based model relies on two crucial aspects: the agents, (their type as well as the interactions between these), and the “world' which hosts the agents.

### Agent implementation

**Figure 1:**
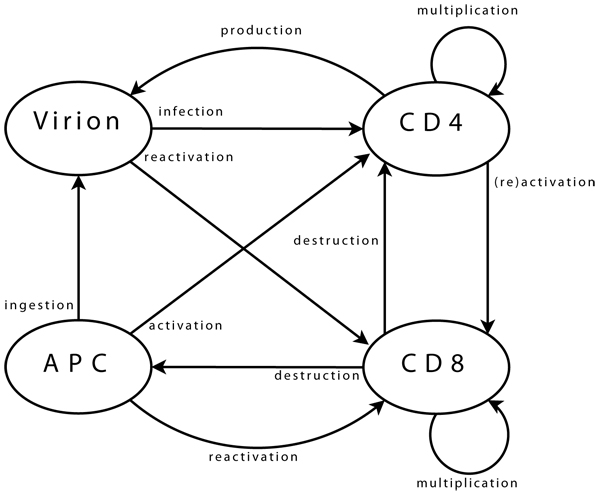
**Agent interactions Viral agents** can move from one neighbourhood to the next, (providing there is sufficient space for them, which is checked using a dedicated function). Upon arrival in a new neighbourhood, these agents can perform a single operation only: infection of a CD4 agent. First step is the selection of CD4 target. Possibility of infection is then assessed, (e.g. if CD4 agent is activated). Infection, if it takes place, is implemented as transfer of viral genome information into the CD4 agent and destruction of viral agent. **CD4 agents** incorporate mobility and can reach new neighbourhoods. As for viral agents, this includes checking, through a dedicated function, that space is available. Upon arrival and if already activated, the agent can activate a CD8 neighbour. Activation follows a process similar to that of infection, detailed above. A possible target is selected, assessed, (in terms of agents bearing “compatible” clonotypes), and then activated when this is possible. If a CD4 agent is infected, it may produce a new viral agent. In the early stages after its own activation, an agent also produces some additional CD4 agents, to enhance immune response. **CD8 agent** mobility is implemented similarly. In its new neigbourhood, the agent produces new CD8 agents, (if it is currently multiplicating), or targets infected CD4 and APC agents, (if it is activated). Some CD8 agents can enter a state representing memory cells. These agents, (with a greater life span and faster reactivation), interact with all agent types in their neighbourhood, in order to monitor known viral strains, and can be directly reactivated. Final agent type, **APC**, implements mobility and associated functions needed to query the environment. APC agents interact with viral agents, in the sense that they can ingest them to present viral strain information to other agents. They also interact with CD4 agents, which they can try to activate by presenting viral information. Success of activation is based on affinity between viral epitope and CD4 clonotype.

In this model, we implement four agent populations which correspond to CD4 and CD8 cells, antigen-presenting cells, and virions, as previously detailed, (see e.g. [19]). Each agent type corresponds to a specific class in *C++*, and inherits from a common, abstract base class. This class contains attributes and functions needed for management of ageing and of location within matrices. At the start of a simulation, initial agent populations are specified in a parameterisation file. Agent populations then dynamically evolve, following rules summarised in Table 2. The reciprocity between agents and biological entities, (cells and virions), mirrors real-system interactions between the latter. These are summarised in Figure 1. To implement these interactions, each agent is assigned an objective:

**Table 2:** Agent population dynamics

Variations	Viral agents	CD4 agents	CD8 agents	APC agents
Increases	Production by infected CD4	Created in thymus, or produced by multiplicating agent	Created in thymus, or produced by multiplicating agent	New agent created
Decreases	Agent ingested by APC, or infecting CD4	Infected agent destroyed, or end of life	End of immune response, or end of life	Presenting agent destroyed, or end of life

At the start of a simulation, initial agent populations are specified in a parameterisation file. Agent populations then dynamically evolve, following specific rules as when agents may be created or destroyed.

• **Viral agents.** These agents have a single and permanent objective: infecting a CD4 agent. In the biological system, the aim is then to produce new virions, (implemented directly within CD4 agents).

• **CD4 agents.** As long as a CD4 agent is neither activated nor infected, its single objective is to stay on “stand-by”, ready to initiate immune responses. This is the initial objective and, since it does not correspond to any particular action, it can be limited to moving within the environment. The objective of an activated agent is to activate CD8 agents, while that of an infected agent is to produce new viral agents. These two objectives can, of course, coexist. The task of a CD4 agent which assumes the task of maintaining memory is similar to that of its initial state.

• **CD8 agents.** As long as a CD8 agent remains non-activated, its single objective is similar to that of initial CD4 agents, and is implemented in the same way. The case of a memory CD8 agent is similar. The task of an activated CD8 agent is to multiply, and to eliminate target agents.

• **APC agents.** An APC has two objectives: locating viral agents, and activating themselves in response to these. The former is the initial state, and is similar to that of other immune agents. The latter can treated as is proposed for CD4 agents activating CD8.

#### *Base class*

Base class attributes are related to the management of agent location and age. Direct implementation of the age would be ill-advised, as it would require updating age of all agents at each iteration, even when this information is not immediately required. This can be quite slow, especially as the number of agents increases.

A more suitable alternative is to save the number of the iteration at which the agent was created. No repetitive update is required, and calculation of the difference between the current iteration number and the “birth date” of the agent provides its age when needed.

#### *Viral agent class*

The viral agent class manages viral strain information. Methods are, therefore, implemented to access and update this information. Having different viral strains means different properties are needed for the associated viral agents: these are not recognised by same set of immune agents, may have distinct mutation rates, etc. Explicitly implementing all these properties within each agent would make them too “large”, in terms of memory usage, (which is to be avoided for large simulations). The solution here is to use a single integer to code the viral strain. This identification can then be used to access strain-specific properties, which are stored in a large array, (representing tens of thousands of potential strains).

Identification of which immune clonotypes recognise each strain is not limited to lock-and-key concepts. Two list of clonotypes are used for each agent. The first list corresponds to clonotypes for which recognition is certain, (i.e. *p* = 1), and the second accounts for those for which recognition is not perfect, (i.e. *p* < 1). These lists can be updated by the agents over the course of the simulation, introducing some *adaptability*. Infection of immune cells is implemented within CD4 agents. As this interaction involves only these two classes, this is biologically equivalent, but it is more efficient, in terms of computing performance.

#### *CD4 and CD8 agent classes*

**Commonalities.** Class attributes are related to management of the class-specific parameters that make up the internal state of these agents: agent clonotype, multiplication status, activation status, memory status, (and infection status for CD4 agents). Methods are provided, to update or access information, (as for viral agents).

The clonotype is coded as an integer, randomly valued when the agent is created. Activation status is also coded as an integer. This value, set initially to zero, is used to store the identification integer representing the viral strain which led to agent activation. A positive value corresponds to an agent activated to respond to HIV strains, while a negative one is linked with another infection the immune system is currently responding to. This is crucial, as not all immune cells are available for a given response: some are already involved in other responses. It is also important, for CD4 agents, since infection requires that the cell is activated, but the involved response needs not be targeted to HIV.

Multiplication status is an integer, initially set to zero. It is then incremented at each step of the multiplication phase, until it reaches a limit and is set back to zero, signalling the end of this phase. Memory status is also coded as an integer and initially set to zero. When an agent starts assuming the role of a memory cell, it takes the value previously assigned for activation status, and that activation status is then set back to zero.

**CD4-specific attribute values.** Infection status is coded as an integer, initially set to zero, and storing HIV strain identification. Infection by multiple strains is possible in the real system. This is not, however, considered in the proposed model, as each set of strain properties may implicitly account for several strains in the real system, if these differ in terms of properties which are not explicitly considered here. A possible extension of the model may be to consider multiple infections.

**CD4-specific methods.** Methods are implemented to account for actions related to infection of a CD4 agent. These correspond to assessment of viral presence in the neighbourhood, and transfer of viral content to the infected agent, (which also implements a chance of viral mutation, since this can occur during reverse transcription of the viral RNA). Additional methods are also implemented for these agents, to deal with activation of a CD8 agent. The process is similar that of infection of CD4 by a viral agent: presence of targets is assessed, and activation takes place if possible.

Similarly, other methods account for production of virions by infected CD4 agents. Here, the agent needs access both to the strain properties array and to its environment.

**CD8-specific methods.** Additional methods are also implemented for these agents, to account for elimination of infected agents. The process is similar that of previous interactions: viz. target assessment and elimination where necessary.

#### *APC agent class*

The APC agent class attributes are related to the management of the single parameter that makes up the internal state: the list of presented viral strains. Viral strains are coded using their identification integer. The list is initially empty, and new integers are added as strains are detected by the agent. Methods are provided to access and update this list.

Additional methods account for activation of CD4 agents. The process is similar that of previous interactions, and involves assessment and activation steps.

### Lymph network implementation

In the context of HIV infection progression and the immune response to this, most of the interactions take place within the lymph nodes. As a consequence, our model relies on an explicit implementation of these: the “world' which hosts our agents is a collection of nodes, which are linked through a network whose structure mimics that of the lymph network.

In order to efficiently account for cell interactions and corresponding physical contact, accurate localisation of agents within the lymph node is required, implying division into smaller subunits. A standard approach to spatial representation requirements is to use a 2D or 3D matrix.

Here, matrix elements can be considered to be “physical”, containing several agents of each type, with a limit based on the size of the modelled entity. This implies that all cell interactions will take place within this physical neighbourhood, and considering surrounding matrix elements is not necessary, (contrary to models where matrix elements can only contain one element, where it is necessary to consider Moore or Von Neumann neighbourhoods for interactions). In our model, lymph nodes are, therefore, implemented as 2D matrices where each element represents a physical, 3D, neighbourhood. The typical size for each element is 10^−12^*m*^3^, (assuming a 10^−8^*m*^3^ volume for each lymph node).

Our implementation of these structures is based on static memory allocation. Large arrays of agents, (one for each agent type), are allocated when simulation starts, and store the maximum number of agents which can be present in the whole matrix at the same time. Each matrix element then only stores integers, used as offsets, to locate agents currently held in these arrays. This guarantees optimality of operations such as creation of new agents and agent mobility within a node. Agent mobility between multiple nodes must also be implemented, and this is obtained by linking these nodes on a network structure. A first step is the inclusion of entry and exit points to each matrix. Upon arrival in a node (through an entry point), an agent randomly circulates in its new environment, until it reaches an exit point and travels to another node, (as detailed below). This typically occurs after 20 to 30 hours, (time step: one minute), and the transfer between two neighbouring nodes markedly shorter (~minutes).

As key defense units, lymph nodes are distributed throughout the body. Humans have around 500 lymph nodes, and lymphocytes constantly circulate through these lymph nodes. To guarantee this cell circulation, connectivity is an essential property of the lymph network: a cell newly produced in the thymus must be able to reach any lymph, and efficient immune response implies interactions between nodes, (by means of cell exchanges).

The lymph network, however, is *not* equivalent to a *complete graph*: between any given pair of nodes, there is a path, but not necessarily a direct connection. In contrast, the lymph network is organised as a set of chains. These “clusters” of nodes can be found in the neck, chest, abdomen, underarm, etc.

The circulation of immune cells between nodes is not trivial. Cell migration is non-random [20], and is, in particular, type-dependent: CD4/CD8 ratio is always higher in the recirculated compared to overall population, (and often more than twice as important). Interestingly, however, transit kinetics are equivalent for both subsets of T cells. Naive and memory cells also have different recirculation pathways [21].

Circulation is also tissue-specific, in the sense that T lymphocytes preferentially recirculate back to the tissues they came from. This is controlled at the molecular-level through specific chemokines [22].

It is important to account for lymph network structure: where this, (as mentioned earlier), is similar to a directed connected graph, but is not complete. It implies that the final destination of an agent leaving a node can theoretically be any node, but its immediate destination is limited to a small subset of nodes. In our model, selection of the final destination is random, but not based on a uniform distribution. To account for preferential recirculation back to tissues of immune cell origin, each lymphocyte agent leaving a node has a 0.5 probability to be assigned this node as its final destination, ensuring circulation through the whole lymph chain corresponding to this node. To respect the higher lymph node to overall population CD4/CD8 ratio in departing agents, a function is also added to guarantee that not all CD8 agents evolving in the neighbourhood of the exit point are selected for departure. More refined implementations exist, (see e.g. [23]), and such an approach may be incorporated in our model at a later stage.

**Figure 2:**
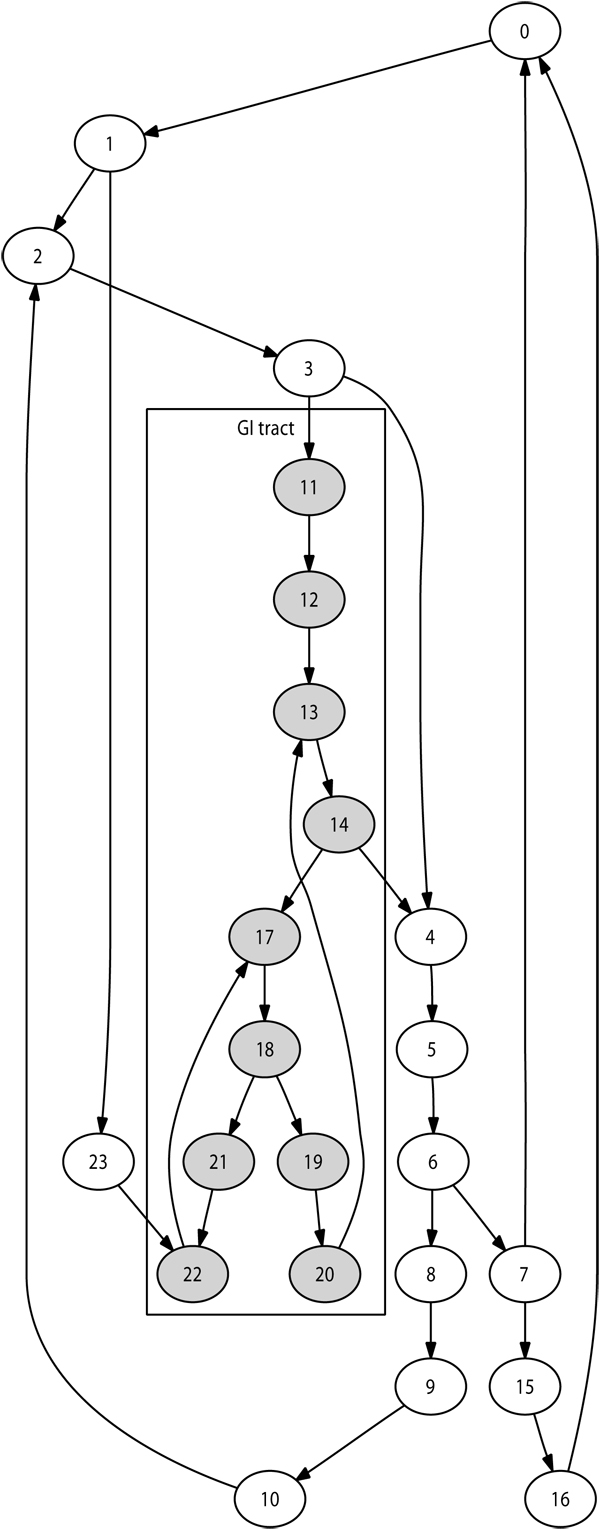
**Simulated 24-node lymph network** Example of a typical lymph network, generated here with 24 nodes. Nodes colored in grey represent the gastro-intestinal tract. Agents located in these nodes are initialised with specific properties associated with this area.

Selection of the immediate destination is based on the lymph network structure modelled. The number of lymph nodes modelled during a given simulation may vary, for instance depending on available computing resources. We implemented an automated technique to generate a network of nodes reproducing the lymphatic chain structures for any number of nodes. This guarantees a anatomically realistic network structure for any simulation size. Figure 2 illustrates this with a 24-node network.

To generate a network with *n* lymph nodes, this technique works as follows:

Create main-chain of user-defined length (e.g. n/4)

Do

Select a node already affected

**If** it is not suitable (e.g. degree to high)

**then** select a neighbor

**End**

Selected node becomes start of lymph chain

Select length for lymph chain

Create lymph chain using nodes not yet affected

Select a node already affected (same technique as above)

Selected node used to close chain

**Until** all nodes are affected

### Parallelisation and GI tract implementation

The system under investigation has a strong parallel nature, in the sense that each node is largely independent of the others. There is no direct interaction between cells located in separate lymph nodes, since cell-level interactions require physical proximity. The consequence is that, *apart from cell transfer,* each node is independent of the others. A node influences its neighbours solely through cells exiting their current location and reaching the next node.

We can, therefore, use a *spatial parallelisation*, based on a reciprocity between the lymph nodes and the computer nodes of a cluster. Each lymph node of the model is assigned to a computer node of the parallel architecture, and the communication network is designed to mimic cell mobility along the lymph network. The tests carried out to evaluate several communication strategies are detailed in [24,25].

With this parallel implementation, it is possible to run simulations involving several hundred lymph nodes, and more than one billion agents. This scale permits the definition of localised subgroups of nodes with specific features.

In an agent-based model, entities update their internal state based on interactions with other agents, but also with the environment itself. It is, therefore, possible to create *compartmental properties* which will locally alter agent behaviour.

Explicit implementation of the lymph nodes is, in that sense, crucial. Since each node is modelled separately, it is possible to select a subset of them and add additional properties for agents in those nodes. For instance, a higher probability of activation by some foreign antigen, not related to HIV, can be specified. Here, we do not distinguish between node types at the structural level, but rather in terms of properties of the cells they host. Providing this can be supported by enough data, later model versions may also include physical differences between lymph nodes.

Thus implementation of the gastrointestinal tract is currently obtained through selection of a long lymph chain in the lymph network structure and alteration of local properties:

• Selected nodes are initialised with agent populations reflecting known cell properties, e.g. 90% of memory cells, most of them active.

• Agent counts in these nodes are initialised to high values, so that these account for half of the overall agent population.

• The probability, for a non-active CD4 or CD8 agent, to be activated by a foreign antigen not related to HIV is increased, so as to maintain overall levels mentioned above.

• The input of “new” cells in those nodes during the simulation is similarly altered.

## Competing interests

The authors declare no competing interest.

## Authors contributions

All authors participated in study design and manuscript preparation. DP implemented and parallelised the model.
